# The contribution of iron deficiency to the risk of peripartum transfusion: a retrospective case control study

**DOI:** 10.1186/s12884-020-02886-z

**Published:** 2020-04-06

**Authors:** H. VanderMeulen, R. Strauss, Y. Lin, A. McLeod, J. Barrett, M. Sholzberg, J. Callum

**Affiliations:** 1 Department of Medicine, Division of Hematology, University of Toronto, Toronto, Canada; 2 Department of Laboratory Medicine and Molecular Diagnostics, Sunnybrook Health Sciences Centre, Toronto, Canada; 3 Department of Laboratory Medicine and Pathobiology, University of Toronto, Toronto, Canada; 4 Department of Medicine, Sunnybrook Health Sciences Centre, Toronto, Canada; 5 Department of Obstetrics and Gynecology, Sunnybrook Health Sciences Centre, Toronto, Canada; 6 Li Ka Shing Knowledge Institute, Toronto, Canada; 7 Department of Medicine, St. Michael’s Hospital, Toronto, Canada

**Keywords:** Pregnancy, Iron deficiency, Anemia, Transfusion

## Abstract

**Background:**

Iron deficiency in pregnancy is associated with inferior maternal and fetal outcomes. Postpartum depression, prematurity, intrauterine growth restriction, impaired childhood cognition and transfusion are all sequelae of maternal iron deficiency anemia. Transfusion to women of childbearing age has important consequences including increasing the risk of hemolytic disease of the fetus and newborn with future pregnancies. The relative contribution of iron deficiency to transfusion rates in the peripartum period is unknown. This study aimed to identify the prevalence of iron deficiency and anemia in pregnant women that received peripartum transfusions relative to age-matched non-transfused controls.

**Methods:**

We performed a retrospective case-control study of all women that were transfused in the peripartum period from January, 2014 to July, 2018. Cases were compared to the next age matched control to deliver at our institution. The primary objective was to determine the proportion of patients with iron deficiency in pregnancy or anemia in pregnancy in cases and controls. Charts were reviewed for predisposing risk factors for iron deficiency, laboratory measures of iron deficiency and anemia, iron supplementation history and maternal and fetal outcomes. Factors associated with peripartum transfusion were analyzed using a multivariate logistic regression.

**Results:**

169 of 18, 294 (0.9%) women were transfused in the peripartum period and 64 (44%) of those transfused received 1 unit. Iron deficiency or anemia were present in 103 (71%) transfused women and 74 (51%) control women in pregnancy (OR 2.34, 95% CI: 3.7–18.0). Multivariate analysis identified social work involvement (adjusted OR 4.1, 95% CI: 1.8–10.1), intravenous iron supplementation in pregnancy (adjusted OR 3.8, 95% CI: 1.2–17.4) and delivery by unscheduled cesarean section (adjusted OR 2.8, 95% CI: 1.3–6.2) as significant predictors of peripartum transfusion.

**Conclusions:**

Pregnant women being followed by a social worker, receiving intravenous iron supplementation in pregnancy or who deliver by unscheduled cesarean section are more likely to receive a red blood cell transfusion. Women with iron deficiency or anemia in pregnancy are at increased risk of peripartum blood transfusions and warrant early and rigorous iron supplementation.

## Background

A drop in hemoglobin (Hb) level is a physiologic consequence of pregnancy due to an expanded plasma volume. The maternal serum hemoglobin typically reaches the physiological nadir at 24–32 weeks gestation [1]. Iron requirements steadily rise in each trimester, reaching a peak of 7.5 mg/d in the 3rd trimester [1]. Overall, pregnancy has a net iron loss of approximately 740 mg [2]. In Canada, 23% of pregnant women are anemic (Hb < 110 g/L in 1st and 3rd trimesters, < 105 g/L in 2nd trimester) [3, 4] and an estimated 85% of these cases are attributable to iron deficiency [5]. In its early stages, iron deficiency can occur without anemia. Ferritin is the best marker of iron deficiency in pregnancy and a serum ferritin less than 30 μg/L has a sensitivity of 92% for iron deficiency with a positive predictive value of 83% [6]. Recent Canadian data showed that over 73% of pregnant women had ferritin levels < 30 μg/L at first obstetrical evaluation [7].

Maternal health is impacted by anemia during pregnancy. A recent analysis describes a 2.36 fold higher risk of maternal death in women with severe anemia (hemoglobin < 70 g/L) [8]. The risk of postpartum depression is also higher in women with iron deficiency anemia (IDA) [9]. Given iron’s critical role in DNA synthesis and cellular metabolism [10], it is not surprising that it also plays a crucial role in fetal development. In fact, maternal IDA increases the risk of prematurity [11, 12], fetal intrauterine growth restriction [11, 13], developmental delay [14] and perinatal mortality [12, 13]. The risks associated with anemia in pregnancy correlate best with hemoglobin levels tested in the first trimester, prior to significant plasma expansion [15].

In addition to these adverse outcomes, treating iron deficiency can also prevent red blood cell (RBC) transfusions for severe, symptomatic anemia. Avoiding RBC transfusions in pregnant women and women of childbearing age is key to preventing the development of red blood cell alloantibodies and hemolytic disease of the fetus and newborn in subsequent pregnancies [16], as one in fifteen recipients of RBC transfusions will develop an alloantibody [17]. Further, any transfusion carries the risk of identification errors, viral or bacterial infection, transfusion associated circulatory overload or other transfusion reactions [18].

Given the high prevalence of iron deficiency in pregnant women, our goal was to elucidate the relative contribution of untreated iron deficiency to peripartum RBC transfusions. We present a case control study aimed at identifying the prevalence of iron deficiency or anemia in pregnant women that were transfused in the peripartum period compared to controls.

## Methods

A retrospective case-control study was carried out with cases defined as all obstetric patients receiving a peripartum RBC transfusion between January, 2014 and July, 2018 during admission to the obstetrical unit at Sunnybrook Health Sciences Centre, Toronto, Canada. High risk referrals comprise 20% of the approximately 4000 deliveries per year at our institution. Cases were identified through the blood bank information system (HCLL, Wellsky, USA). Patients were excluded if they had a clear alternative explanation for their anemia (e.g., chronic kidney disease, malignancy, bone marrow failure syndrome), had a pre-existing transfusion dependence, had differential hemoglobin targets for transfusion (e.g., sickle cell disease), or did not receive the majority of their prenatal care at our institution (defined as women whose care was transferred to our institution for delivery only). Controls were defined as obstetrical patients delivering at our institution that did not receive a transfusion at the time of delivery. A single control was selected as the next age-matched delivery following the transfused case.

Demographic and laboratory data were collected from patient charts retrospectively. Transfusion data were collected from the blood bank information system. Based on the literature and biologic plausibility, risk factors for and laboratory measures associated with iron deficiency, anemia and transfusion were established [19, 20]. Demographic variables collected included age, ethnicity, multiple gestation, social work involvement, history of heavy menstrual bleeding, history of chronic malabsorption (e.g. celiac disease, inflammatory bowel disease, bypass surgery), vegetarianism/veganism, hyperemesis gravidarum, and consumption of prenatal vitamins and iron supplements during pregnancy. Patients’ past medical history, including obstetrical and gynecologic history, and dietary restrictions were collected from their prenatal record. This record is standard and mandated for all pregnancies in the province of Ontario, Canada. Median household income was assigned based on geographic location of residence by postal code using publicly available records from Statistics Canada (Ottawa, Canada). Delivery outcomes and perinatal outcomes were also collected including incidence of primary postpartum hemorrhage (defined by > 1000 mL of blood loss within 24 h of delivery as estimated on their delivery record, or documentation of postpartum hemorrhage in the chart), maternal infection, fetal demise, prematurity (< 37 weeks gestational age), low birth weight (< 2500 g), maternal ICU admissions, hysterectomies and cesarean sections (planned and unplanned). We collected components of the complete blood count (hemoglobin, mean corpuscular volume (MCV), red blood cell distribution width (RDW)) and iron studies (ferritin, transferrin saturation (TSAT) and total iron binding capacity (TIBC)) in the year prior to pregnancy, during pregnancy and up to 6 months postpartum.

The primary objective was to determine the proportion of patients with iron deficiency in pregnancy (ferritin < 30 μg/L, TSAT < 20% or last documented MCV < 80 fL when previously normal) or anemia in pregnancy (Hb < 110 g/L in 1st or 3rd trimester or < 105 g/L in 2nd trimester) in cases and controls.

The secondary objectives were to determine the rate of suboptimal care as defined by any of the following quality indicators: 1) Unaddressed anemia in pregnancy (defined by anemia (as above) without either the measurement of a ferritin or transferrin saturation and total iron binding capacity, or without iron treatment documented in the antepartum record; and absence of a known red cell disorder as documented in laboratory information system or antenatal record. The standard of care in Canada is to offer hemoglobin electrophoresis or high performance liquid chromatography to at-risk women [21]); 2) Identified but uncorrected iron deficiency in pregnancy: clear iron deficiency (ferritin < 30 μg/L) without documentation of commencing oral iron replacement at a dose of at least 30 mg of elemental iron or administration of intravenous iron; 3) Identified but uncorrected iron deficiency anemia in pregnancy: clear iron deficiency anemia (ferritin < 30 μg/L and anemia (defined above)) without documentation of commencing oral iron replacement at a dose of at least 30 mg of elemental iron or administration of intravenous iron; 4) Mild anemia (Hb 100–109 g/L in 1st and 3rd trimester, Hb 100–104 g/L in 2nd trimester) and moderate anemia (Hb 90–99 g/L) in pregnancy; and 5) Hb < 90 g/L postpartum with uncorrected iron deficiency (defined above) at last measurement in the current pregnancy.

Descriptive statistics were summarized using medians and interquartile ranges for continuous variables and proportions for categorical variables. To identify statistically significant predictive factors associated with peripartum transfusion, univariate logistic regression was performed with case/control status as the dependent variable. We included predefined risk factors for iron deficiency, anemia and transfusion which were selected based on biologic plausibility and sufficient data. Statistically significant variables at a threshold of alpha 0.05 were included in a multivariate logistic regression model to quantify predictive factors associated with transfusion at delivery. Maternal and fetal outcomes were excluded from our multivariate analysis in order to focus on preexisting risk factors for transfusion. Odds ratio (OR) and the 95% confidence interval (CI) were calculated for each predictive factor. All statistical analyses were performed using Statistical Analysis Software (SAS version 9.4; Cary, NC).

This study was approved by the Sunnybrook Health Sciences Centre Research Ethics Board, #221–2018; this approval allowed access to the data used for this research.

## Results

### Demographics

Between January, 2014 and July, 2018, 18, 294 women delivered at our institution. Of those, 169 (0.9%) women received an RBC transfusion in pregnancy or up to 6 weeks postpartum. Twenty four cases did not meet the inclusion criteria: received the majority of their care at other institutions (missing baseline laboratory data) [7], chronic kidney disease [7], sickle cell disease [4], aplastic anemia [2], rectal cancer [1], B cell acute lymphoblastic leukemia [1], pre-existing transfusion dependence due to unknown hematologic disorder [1] and a planned open hysterectomy at the time of delivery for uterine fibroids [1]. After excluding these 24 women, 145 cases remained and were matched to controls, resulting in an overall cohort size of 290 patients.

The mean patient age was similar in both groups (34.5 ± 5.0) (mean age of 33.1 for all deliveries at this centre during time period). Baseline characteristics of the population are presented in Table 1. Transfused cases as compared to controls were more likely to be primiparous (20% vs 15%), to have non-singleton pregnancies (8% vs 4%) and to be delivered by cesarean section (60% vs 38%).

**Table 1:** Patient demographics for cases and controls

Demographics		Case (*N* = 145)	Control (*N* = 145)
Age – mean (standard deviation)		34.5 (±5.0)	34.5 (±5.0)
Primiparous – n (%)		29 (20)	22 (15)
Caucasian – n (%)		13 (24)	42 (47)
English as primary language – n (%)		73 (90)	118 (96)
Median household income ($ CAD) – median (Q1-Q3)		92,160 (72453–115,438)	94,254 (77846–119,354)
Vegetarian – n (%)		6 (4)	2 (1)
Prenatal vitamin – n (%)		136 (94)	143 (99)
Mode of delivery – n (%)	Vaginal:	58 (40)	90 (62)
	Cesarean:	87 (60)	55 (38)

### Transfusion metrics

**Fig. 1:**
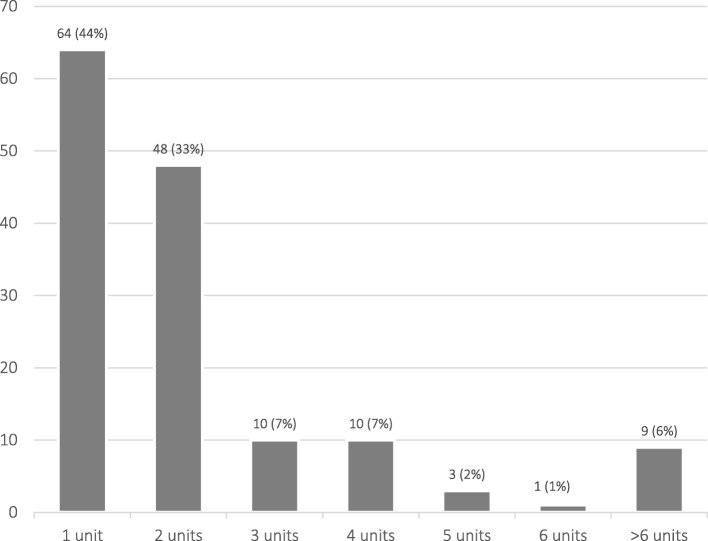
Number of units of red blood cells transfused per patient (*N* = 145 patients)

**Fig. 2:**
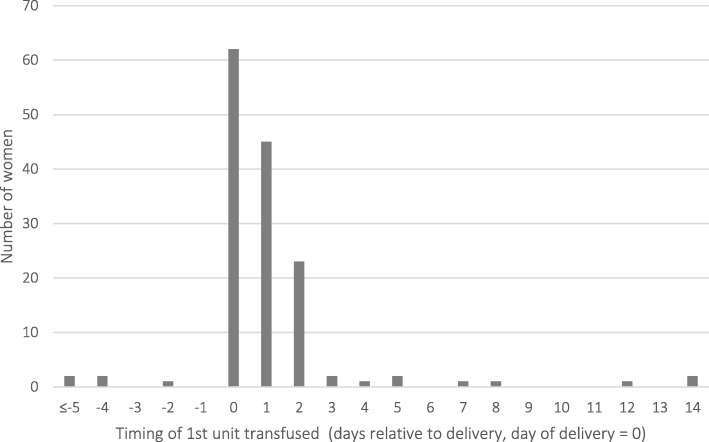
Timing of peripartum red blood cell transfusions (*N* = 145 patients)

A total of 431 units of RBCs were transfused to 169 women (72 units were transfused to the 24 excluded patients and 359 to the 145 cases). For the 145 cases, 64 (44%) women received 1 unit of red blood cells, 48 (33%) received 2 units, and 33 (23%) women required 3 or more units (Fig. 1). Four women were transfused antepartum and 141 were transfused at delivery or postpartum (Fig. 2). The median pre-transfusion hemoglobin was 65 g/L (IQR 11.25). Ninety-four of the 145 cases had a document of postpartum hemorrhage on their record and 15 were transfused as part of a massive transfusion protocol. In contrast, only 4 controls had documented postpartum hemorrhage.

### Primary outcome

Iron deficiency or anemia were present in 103 (71%) transfused women and 74 (51%) control women in pregnancy (OR 2.34, 95% CI: 1.4–3.8). Secondary outcomes are reported in Table 2. The odds of moderate postpartum anemia (Hb < 90 g/L) with uncorrected iron deficiency in pregnancy (OR 7.7, 95% CI: 3.7–18.0), moderate anemia in pregnancy (OR 5.2, 95% CI: 1.1–49.9) and mild anemia in pregnancy (OR 3.2, 95% CI: 1.8–6.2) were significantly higher in transfused women relative to controls.

**Table 2:** Secondary outcomes: rates of suboptimal care in case vs. control patients based on a priori criteria

Metric	N case; N control	Case	Control	Odds ratio (95% CI)
Postpartum Hb < 90 g/L with uncorrected antepartum iron deficiency - n (%)	144, 85	66 (46)	8 (9)	7.7 (3.7–18.0)
Moderate anemia in pregnancy (Hb 90–99 g/L) - n (%)	145; 145	7 (5)	1 (0.7)	5.2 (1.1–49.9)
Mild anemia in pregnancy (1st + 3rd trimester Hb 100–109 g/L, 2nd trimester Hb 100–104 g/L) - n (%)	145; 145	42 (29)	16 (11)	3.2 (1.8–6.2)
Identified and uncorrected iron deficiency anemia in pregnancy (anemia and ferritin < 30 μg/L without documentation of oral or intravenous iron replacement) - n (%)	101; 102	11 (11)	5 (5)	2.2 (0.8–6.7)
Unaddressed anemia in pregnancy - n (%)	145; 145	16 (11)	8 (6)	2.1 (0.9–5.1)
Identified and uncorrected iron deficiency in pregnancy (ferritin < 30 μg/L without documentation of oral or intravenous iron replacement) - n (%)	101; 102	36 (36)	35 (34)	1.0 (0.6–1.8)

*Hb* hemoglobin

### Iron status

**Fig. 3:**
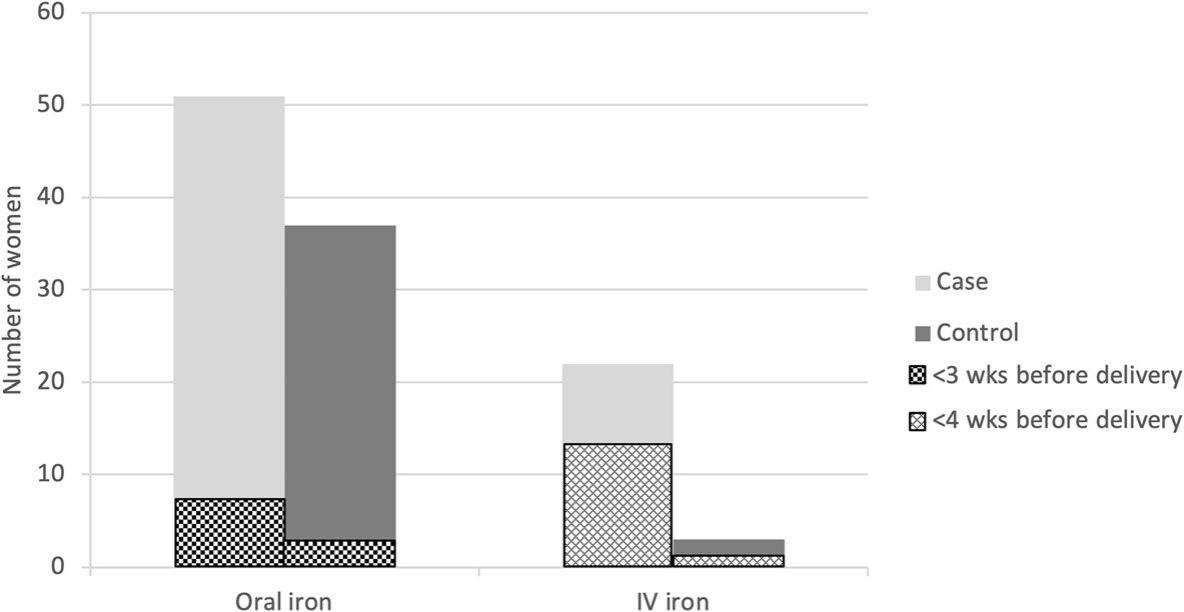
Use of and timing of oral and intravenous iron supplementation in pregnancy (*N* = 145 cases, *N* = 145 controls)

Iron deficiency was identified in 27 of 85 cases (32%) and 9 of 41 controls (22%) in the year prior to pregnancy. In pregnancy, 72 cases (50%) and 62 controls (43%) had iron deficiency, and 32 cases (22%) and 11 controls (8%) had iron deficiency anemia. Iron deficiency was identified in 14 of 144 cases (10%) and 5 of 75 controls (7%) who had a CBC, TSAT, and/or ferritin in the period from 24 h postpartum to 6 months postpartum (Table 3). Fifty-one (35%) transfused women received oral iron replacement in pregnancy compared to 37 (26%) women in the control group. Of these 88 women treated with oral iron, 7 transfused cases and 1 control case were started on oral iron within 4 weeks of delivery. Twenty-two (15%) transfused women and 3 (2%) control women received intravenous iron therapy in pregnancy. Iron sucrose was the formulation used in all cases at a dose of 200–300 mg IV per infusion. Sixteen of the 22 (73%) transfused women who received intravenous iron were given their first dose within 3 weeks of delivery (Fig. 3). Of the transfused women, 7 received 1 dose of intravenous iron in pregnancy, 13 received 2 doses and 2 received 3 doses. In the control group, 1 woman received 2 doses of intravenous iron in pregnancy and 2 women received 3 doses of intravenous iron in pregnancy.

**Table 3:** Laboratory values and iron therapy in cases and controls

Laboratory measure	N case; N control	Case	Control
Ferritin measured in pregnancy – n (%)	145; 145	101 (70)	102 (70)
Ferritin measured in 1st trimester – n (%)	145; 145	56 (39)	56 (39)
Preconception iron deficiency – n (%)	85; 41	27 (32)	9 (22)
Ferritin < 30 μg/L – n (%)	53; 23	26 (49)	8 (35)
TSAT < 20% – n (%)	2; 1	0 (0)	0 (0)
MCV < 80 fL when previously normal– n (%)	85; 40	2 (2)	1 (3)
Preconception anemia – n (%)	85; 40	15 (18)	8 (20)
Iron deficiency in pregnancy – n (%)	145; 145	72 (50)	62 (43)
Ferritin < 30 μg/L – n (%)	101; 102	64 (63)	59 (58)
TSAT < 20% – n (%)	2; 2	2 (100)	2 (100)
MCV < 80 fL when previously normal – n (%)	145; 131	8 (6)	8 (6)
Anemia in pregnancy – n (%)	145; 145	63 (44)	23 (16)
Iron deficiency anemia in pregnancy – n (%)	145; 145	32 (22)	11 (8)
Lowest hemoglobin in pregnancy - median in g/L (IQR)	145; 145	108 (14)	117 (12)
Lowest ferritin in pregnancy – median in μg/L (IQR)	101; 102	24 (24)	27 (30)
Lowest MCV in pregnancy – median in fL (IQR)	145; 145	87.6 (6.5)	88.2 (5.9)
Postpartum iron deficiency – n (%)	144; 75	14 (10)	5 (7)
Ferritin < 30 μg/L – n (%)	20; 7	7 (35)	0 (0)
TSAT < 20% – n (%)	1; 0	0 (0)	0 (0)
MCV < 80 fL when previously normal – n (%)	144; 74	7 (5)	5 (7)
Postpartum anemia (Hb < 110 g/L up to 6 months)– n (%)	144; 74	142 (99)	39 (53)
Oral iron – n (%)	145; 145	30 (42)	23 (37)
Intravenous iron – n (%)	145; 145	22 (15)	3 (5)

*IQR* interquartile range*, MCV* mean corpuscular volume*, TSAT* transferrin saturation

### Univariate analysis

Results of the univariate regression analysis are detailed in Table 4. Socioethnic factors including Caucasian ethnicity (OR 0.4, 95% CI: 0.2–0.7), social work involvement during pregnancy (OR 5.0, 95% CI: 2.4–11.7) and residency in a low-income neighborhood (OR 2.0, 95% CI: 1.2–3.5) had significant associations with the odds of transfusion. Women with anemia of any etiology in pregnancy had 4 times higher odds of receiving a transfusion (OR 4.0, 95% CI: 2.3–7.1). Transfused women had higher rates of IDA in pregnancy (OR 3.3, 95% CI: 1.7–7.1). Vaginal delivery was associated with lower odds of transfusion (OR 0.4, 95% CI: 0.3–0.7), while delivery by unplanned cesarean section was associated with higher odds (OR 3.7, 95% CI: 2.2–6.4).

**Table 4:** Univariate analysis: factors associated with increased risk of transfusion and maternofetal outcomes

	N case; N control	Case	Control	Odds Ratio (95% CI)
Baseline characteristics				
IV iron supplementation in pregnancy – n (%)	145; 145	22 (15)	3 (2)	7.4 (2.6–28.3)
Social work involvement – n (%)	145; 145	34 (23)	8 (6)	5.0 (2.4–11.7)
Unplanned cesarean section – n (%)	145; 145	67 (46)	27 (19)	3.7 (2.2–6.4)
Iron deficiency anemia in pregnancy – n (%)	145; 145	32 (22)	11 (8)	3.3 (1.7–7.1)
Residence in lowest quartile income neighborhood – n (%)	144; 145	44 (31)	26 (18)	2.0 (1.2–3.5)
Highest RDW in pregnancy - median (IQR)	144; 145	14.3 (1.5)	13.9 (1.2)	1.3 (1.1–1.5)
Caucasian – n (%)	55; 90	13 (24)	42 (47)	0.4 (0.2–0.7)
Vaginal delivery – n (%)	145; 145	58 (40)	90 (62)	0.4 (0.3–0.7)
Twins/triplets – n (%)	145; 145	12 (8)	6 (4)	2.0 (0.8–5.7)
Pre-conception iron deficiency – n (%)	85; 41	27 (32)	9 (22)	1.5 (0.7–3.7)
Planned cesarean section – n (%)	145; 145	20 (14)	28 (19)	0.7 (0.4–1.3)
Outcomes				
Primary postpartum hemorrhage – n (%)	145; 145	94 (65)	4 (3)	65.0 (22.7–185.8)
Maternal bacterial infection – n (%)	145; 145	22 (15)	1 (1)	25.8 (3.4–193.8)
Fetal demise – n (%)	145; 145	17 (12)	2 (1)	9.5 (2.2–41.9)
Prematurity (< 37 weeks) – n (%)	145; 145	68 (47)	14 (10)	8.3 (4.4–15.7)
Low birth weight (< 2500 g) – n (%)	145; 145	64 (44)	15 (10)	6.8 (3.6–12.8)
NICU admission – n (%)	145; 145	51 (35)	13 (9)	5.5 (2.8–10.7)
Maternal ICU admission – n (%)	145; 145	19 (13)	0 (0)	NA
Hysterectomy – n (%)	145; 145	13 (9)	0 (0)	NA

*ICU* intensive care unit*, NA* not available*, NICU* neonatal intensive care unit*, IQR* interquartile range

### Multivariate analysis

The multivariate analysis included all statistically significant baseline predictors of transfusion from the univariate analysis (Table 5). Social work involvement in pregnancy (OR 4.1, 95% CI: 1.8–10.1), IV iron supplementation in pregnancy (OR 3.8, 95% CI: 1.2–17.4) and delivery by unplanned cesarean section (OR 2.8, 95% CI: 1.3–6.2) were independently associated with higher odds of peripartum transfusion.

**Table 5:** Multivariate analysis: baseline factors associated with increased risk of transfusion

	N case; N control	Case	Control	Odds Ratio (95% CI)
Social work involvement – n (%)	145; 145	34 (23)	8 (6)	4.1 (1.8–10.1)
IV iron supplementation in pregnancy – n (%)	145; 145	22 (15)	3 (2)	3.8 (1.2–17.4)
Unplanned cesarean section – n (%)	145; 145	67 (46)	27 (19)	2.8 (1.3–6.2)
Iron deficiency anemia in pregnancy – n (%)	145; 145	32 (22)	11 (8)	2.0 (0.9–4.6)
Residence in lowest quartile income neighborhood – n (%)	144; 145	44 (31)	26 (18)	1.5 (0.8–2.9)
Highest RDW in pregnancy - median (IQR)	144; 145	14.3 (1.5)	13.9 (1.2)	1.2 (0.9–1.4)
Vaginal delivery – n (%)	145; 145	58 (40)	90 (62)	1.0 (0.5–2.0)

*Note: Ethnicity was excluded from multivariate analysis due to small sample size*

*288 patients were included in the multivariate analysis*

### Maternofetal outcomes

Transfused women had more primary postpartum hemorrhage (defined as > 1000 mL blood loss within 24 h of delivery) (OR 65.0, 95% CI: 22.7–185.8) and bacterial infections (OR 25.8, 95% CI: 3.4–193.8). Maternal infections in the transfused women included chorioamnionitis [9], endometritis [6] and surgical site infections [1], among others [4]. In the control group, 1 woman was diagnosed with chorioamnionitis. Babies born to transfused mothers had higher rates of fetal demise (OR 9.5, 95% CI: 2.2–41.9), prematurity (OR 8.3, 95% CI 4.4–15.7), low birth weight (OR 6.8, 95% CI: 3.6–12.8), and NICU admission (OR 5.5, 95% CI: 2.8–10.7) (Table 4).

## Discussion

To our knowledge this is the first study to assess the burden of iron deficiency in women transfused in the peripartum period. We find that transfused women were more than 2-times more likely to have iron deficiency or anemia in pregnancy. Women treated with intravenous iron in pregnancy (OR 3.8), women who delivered by unplanned cesarean section (OR 2.8), and women being followed by a social worker (OR 4.1) were also found to have increased odds of peripartum transfusion. In addition, 77% of women received only 1–2 units of red blood cells. These findings suggest that there is an opportunity to identify and treat iron deficiency before and during pregnancy to mitigate the risk of transfusion and that coordination between physicians and social workers may be a useful strategy to identify and reach women at higher risk.

At our institution, 70% of pregnant women had a ferritin checked and only 65% with documented iron deficiency were prescribed oral iron supplementation. The American College of Obstetricians and Gynecologists (ACOG) [22], Centers for Disease Control (CDC) [23], and World Health Organization (WHO) [4] all recommend screening asymptomatic pregnant women for IDA using serum hemoglobin and ferritin levels, and universal iron supplementation during pregnancy with a dose of 30–60 mg/day of elemental iron [23, 24]. In anemic women, they suggest increasing the dose to 60–120 mg/day of elemental iron [23]. A consensus statement from the Network for the Advancement of Patient Blood Management, Haemostasis and Thrombosis (NATA) suggests daily oral iron (30-60 mg) supplementation in areas with a high prevalence of anemia in pregnancy and screening for iron deficiency in all non-anemic women at risk of ID [25]. The U.S. Preventative Task Force cites a lack of evidence to support routine screening or routine iron supplementation in pregnancy [26]. Perhaps as a result of this ambiguity, less than one third of American obstetrician-gynecologists have been shown to provide routine iron supplementation to their pregnant patients [27]. The NATA consensus statement also supports intravenous iron consideration in women with ID and Hb < 80 g/L or in newly diagnosed IDA after 34 weeks gestation [25]. In our study patients, however, we noted a delay in the initiation of intravenous iron in pregnancy: three-quarters of intravenous iron was given within 3 weeks of delivery, leaving little time for erythropoiesis to correct the red cell mass. The reasons for this delay are likely multifactorial, including a lack of physician comfort with prescribing intravenous iron in pregnancy, difficulty accessing the drug, and challenges in facilitating drug infusion. When possible, we recommend earlier consideration of intravenous iron in women not responding to oral iron, not tolerating oral iron or with moderate to severe anemia to ensure sufficient time to correct the anemia before delivery.

Despite a publicly funded healthcare system in Ontario, oral and intravenous iron are not universally funded as general list products and as such coverage is provided out-of-pocket, through third-party private insurance plans or via exceptional access public funding. However, this does not address the “working poor” who cannot pay out-of-pocket, do not have private insurance and also do not meet criteria for exceptional access public funding. This results in health inequity between high and low income families and likely contributes to the higher incidence of untreated iron deficiency in those of lower socioeconomic status [28]. Moreover, these women are less likely to eat a diet rich in iron as many of these foods are particularly expensive (e.g. red meat). Since children born to iron deficient mothers have higher rates of developmental delay [14] and impaired self-regulation [29], there is a compounding disadvantage of being born into a low-income household. Further, these babies may represent a more medically complex population since in our study, babies born to transfused mothers had higher rates of prematurity and lower birth weights. We also found that Caucasian women had lower peripartum transfusion rates which is in keeping with prior literature [30]. Multiple factors including diet, socioeconomic status and genetics may be at play since Black women have lower hemoglobin levels [31] and non-Caucasians have higher rates of hemoglobinopathies [32, 33]. Further research is required to fully understand this phenomenon and the maternal and fetal benefits of removing barriers to IV iron in pregnancy.

The peripartum transfusion rate at our institution (0.9%) was comparable to that in other high resource settings [19, 20]. Interestingly, 44% of transfused women received just one unit of red blood cells, suggesting that many of the transfusions given were potentially avoidable with diligent prevention of iron deficiency anemia prior to delivery. Avoiding blood transfusions in women of childbearing age is essential to preventing complications such as alloantibody formation and hemolytic disease of the fetus and newborn in future pregnancies. Postpartum transfusion also has other important implications for women: those transfused 1 to 2 units of blood after obstetrical hemorrhage have longer hospital stays, higher ICU admission rates and hospital readmission rates and lower breastfeeding rates [34]. Further, when postpartum women are transfused in the absence of hemorrhage, their risk of severe morbidity with subsequent pregnancies is over four fold [35].

Next steps include clarifying the factors that contribute to higher peripartum transfusion rates in non-Caucasian women and women with complex social situations and quality improvement initiatives to target earlier and more intensive iron replacement in pregnant women. Additional studies need to be performed to understand the proportion of transfusions in these subgroups of patients that can be prevented with oral and intravenous iron. Prospective studies are needed to determine if aggressive iron management in pregnancy will reduce the proportion of women transfused and impact important maternal and fetal outcomes.

### Limitations

First trimester ferritin testing is not part of Canadian obstetrical guidelines and 30% of the patients in this study did not have a ferritin level checked in pregnancy. As such, this study may underestimate the incidence of peripartum iron deficiency. Further, some women received part of their antenatal care at an outside institution and we did not have access to these records or laboratory results for the analysis. As this was a retrospective study, the prescription of oral iron may not be routinely documented in patients’ medication records or clinic notes and therefore may not have been captured.

## Conclusions

In summary, iron deficiency in pregnancy is common, under-recognized and undertreated. It has implications for mothers and babies and predisposes women to blood transfusions which may have severe consequences for future pregnancies. Women being followed by a social worker appear particularly vulnerable and warrant more attention paid to iron replacement and we hypothesize that social and financial barriers may be impairing access to treatment. We advocate for early recognition of iron deficiency by screening all pregnant women with a first trimester ferritin, diligent use of early oral iron replacement, and improved access to intravenous iron replacement in pregnancy for all women irrespective of their financial resources.

## Data Availability

The datasets used and/or analyzed during the current study are available from the corresponding author on reasonable request.

## Abbreviations

Hb: Hemoglobin
IDA: Iron deficiency anemia
MCV: Mean corpuscular volume
RDW: Red blood cell distribution width
TSAT: Transferrin saturation
