# Correction to: The contribution of iron deficiency to the risk of peripartum transfusion: a retrospective case control study

**DOI:** 10.1186/s12884-020-02932-w

**Published:** 2020-04-17

**Authors:** H. VanderMeulen, R. Strauss, Y. Lin, A. McLeod, J. Barrett, M. Sholzberg, J. Callum

**Affiliations:** 1 Department of Medicine, Division of Hematology, University of Toronto, Toronto, Canada; 2 Department of Laboratory Medicine and Molecular Diagnostics, Sunnybrook Health Sciences Centre, Toronto, Canada; 3 Department of Laboratory Medicine and Pathobiology, University of Toronto, Toronto, Canada; 4 Department of Medicine, Sunnybrook Health Sciences Centre, Toronto, Canada; 5 Department of Obstetrics and Gynecology, Sunnybrook Health Sciences Centre, Toronto, Canada; 6 Li Ka Shing Knowledge Institute, Toronto, Canada; 7 Department of Medicine, St. Michael’s Hospital, Toronto, Canada

**Correction to: BMC Pregnancy Childbirth (2020) 20:196**

**https://doi.org/10.1186/s12884-020-02886-z**

Following publication of the original article [1], we have been notified that there is a missing conflict of interest. The Competing interests section is updated as follow:

Dr. Michelle Sholzberg has received honoraria for advisory boards from Pfizer. Dr. Yulia Lin has received research grants from Novartis and consulting fees from Pfizer. The remaining authors declare that they have no competing interests.
